# Supplementary figures and images for: Efficacy of Non-Invasive Brain Stimulation for Refractory Obsessive-Compulsive Disorder: A Meta-Analysis of Randomized Controlled Trials
Source: Brain Sci. 2022 Jul 19;12(7):943. doi: 10.3390/brainsci12070943 (PMC9313124; doi:10.3390/brainsci12070943)

## Supplementary Materials

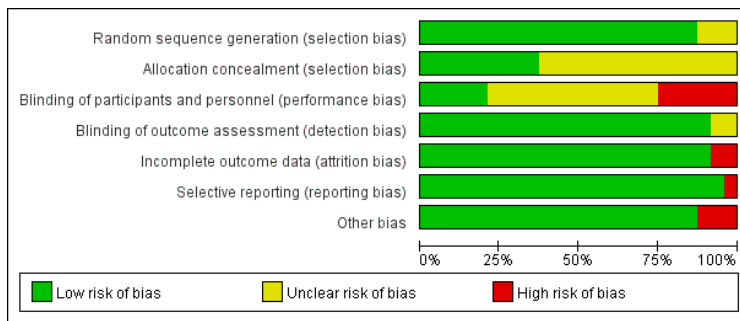

Figure S1. Risk of bias graph.

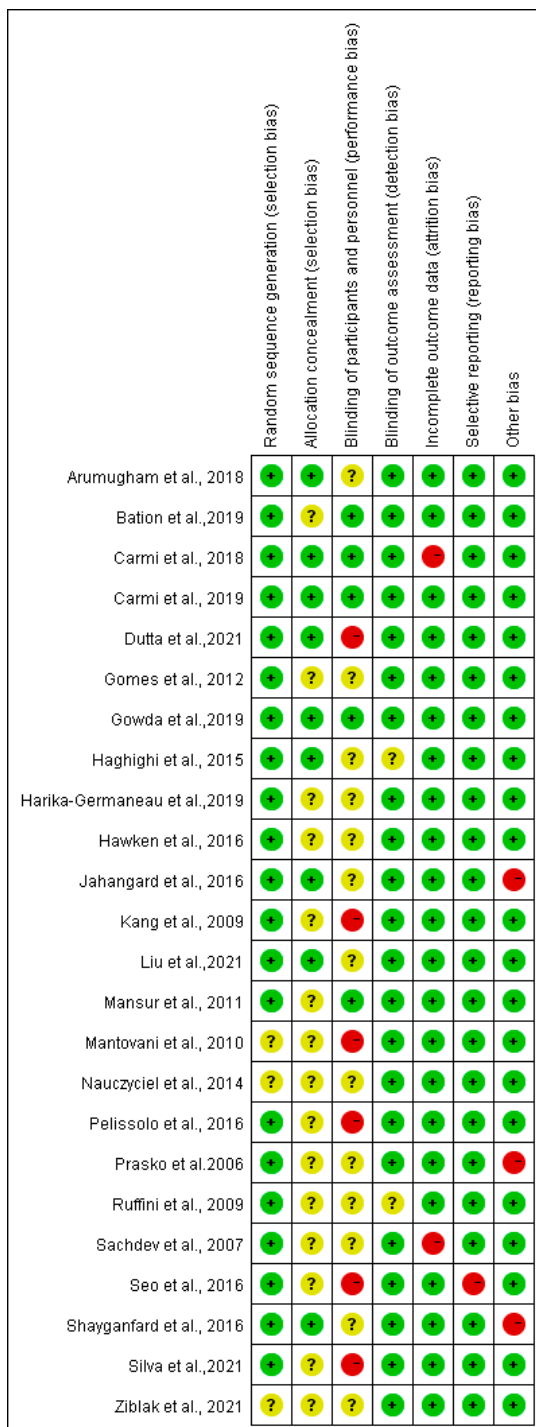

Figure S2. Risk of bias summary.

Supplement: Supplementary file 1 [file brainsci-12-00943-s001.zip › brainsci-1691268-supplementary.pdf]
